# Scaling up a Positive Safety Culture among Construction Small and Medium-Sized Enterprises in Ghana

**DOI:** 10.3390/ijerph21070817

**Published:** 2024-06-22

**Authors:** Eric Adzivor, Fidelis Emuze, Moses Ahiabu, Moses Kusedzi

**Affiliations:** 1 Department of Building Technology, Faculty of Built and Natural Environment, Ho Technical University, Volta Region Box HP 217, Ho, VH-0044-6820, Ghana; 2 Department of Built Environment, Faculty of Engineering, Built Environment and Information Technology, Central University of Technology, Free State, Private Bag X20539, Bloemfontein 9300, South Africa

**Keywords:** accident, construction, culture, safety, Ghana

## Abstract

The Ghanaian construction industry faces challenges in managing safety, especially for small and medium-sized enterprises (SMEs) that need more resources. This research addressed the critical need for a positive safety culture framework specifically designed for SMEs in Ghana. The study adopts the Delphi research approach, which involves a series of questionnaire ‘rounds’ to gather and refine information and develop a collaborative safety culture framework with SME stakeholders. The study employed a mixed-methods strategy, harnessing quantitative and qualitative data to meet the research goals. The critical components of the developed framework included safety commitment, adaptability, information, awareness, culture, and performance. The research offered evidence-based recommendations for effective positive safety practices across Ghana’s SMEs by analysing the relationship between these interventions and safety outcomes. Applying the framework should reduce workplace accidents and foster a positive safety culture that aligns with international best practices.

## 1. Introduction

The assertion that the construction industry in Ghana is a significant contributor to national economic growth with essential health and safety management implications is supported by authors [1], who have discussed the industry’s role in social and economic development in Ghana, as well as the substantial health and safety (H&S) risks associated with construction activities. The composition and dynamics of SMEs in Ghana’s construction industry contributed about 70% of the Gross Domestic Product (GDP) and accounted for approximately 90% of businesses in Ghana [2]. Despite the significant contribution of SME contractors, Ghana’s fatal and non-fatal injury rates or estimates between 2010 and 2016 accounted for over 558 accidents at construction sites [2]. Adopting a safety culture is pivotal to improving health and safety outcomes for small and medium-sized enterprises (SMEs) in this sector. Recent research has indicated a pressing need for frameworks that address the Ghanaian construction industry’s characteristics and challenges to ensure its workforce’s safety. The importance of developing safety culture frameworks for SMEs has been echoed by scholars [2], who have emphasised the need for safety culture indicators to enhance the health and safety performance of SME contractors in Ghana. These frameworks are essential due to the industry’s distinct characteristics and challenges, as elaborated in research which examined health and safety practices in Ghanaian construction SMEs [3].

Building a positive safety culture in SMEs is a complex task, requiring understanding the sector’s specific constraints. These include the need for a skilled and educated workforce, reliance on labour-intensive methods, and the absence of a unified regulatory authority [4,5,6]. These constraints can be addressed through various strategies, such as creating a safety-conscious culture and establishing a funding mechanism for health and safety training [5]. The role of transformational leadership in moderating the relationship between safety culture and safety performance is also crucial [7]. However, it is essential to consider the internal context of each SME and the characteristics that define it, as this can impact the effectiveness of safety interventions [8]. These challenges necessitate strategic interventions that are aware of the industry’s peculiarities. Studies have begun to identify safety culture indicators tailored for SME contractors in Ghana, aiming to enhance health and safety performance and protect construction workers more effectively.

Many studies have highlighted the diversity of health and safety practices in construction SMEs, with many needing to take proactive measures [4,9]. This is often due to financial pressures and a lack of awareness, which are common indicators [10,11]. However, there is a consensus among health and safety experts on leading indicator metrics that could drive positive change in these SMEs [12,13]. These include upper management commitment, worker involvement, and formal and informal communication [12]. The influence of contextual factors, such as the institutional structure and economic climate, on health and safety management has also been noted [9]. Developing a positive safety culture within SMEs in the Ghanaian construction industry should consider these unique attributes and constraints and focus on scaling safety measures that are both effective and feasible within the SME context. By doing so, there is potential to align with international best practices and address Ghana’s local socio-economic and cultural realities. The construction industry in Ghana faces significant health and safety challenges, particularly in SMEs [9]. These challenges are exacerbated by a lack of proactive health and safety practices, a low safety culture maturity level, and a need for more emphasis on safety as a business risk [14]. Ghana’s political and socio-cultural environments also shape SME H&S management in developing economies [15]. The extended family system and collectivist value systems influence safety attitudes and behaviours [14,16]. Despite the presence of health and safety policies, their implementation and functionality often need to be improved [16]. The institutional structure, economic climate, and extended family culture are critical challenges to SMEs’ effective health and safety management [9]. To address these challenges, a safety culture development model is proposed, emphasising the role of construction safety culture in minimising accidents and incidents [17,18]. The importance of a positive safety culture, shaped by common sense and informal practices, is also highlighted [19,20].

The Ghanaian construction industry, particularly SMEs, faces significant challenges in developing a positive safety culture. The need for indicators and assessments to improve safety culture to overcome the industry’s current state at the pathological stage is confirmed [14,21]. Health and safety policies and the influence of the contextual environment cannot be ignored [9,16]. Further, adequate health and safety programmes and the role of safety culture have been highlighted in the literature [22]. A positive safety culture provides specific strategies for curbing workplace accidents, including using incentives and effective communication [23]. These studies underscore the need for a framework to address the safety culture challenges in the Ghanaian construction industry. However, the need for a positive safety culture as an indicator in the tendering evaluation compliance procedures and safety networking among SMEs has been a missing link within the Ghanaian construction SMEs. Building a positive safety culture within SMEs is a complex task that requires understanding the specific constraints within the sector [17]. These constraints emanate from lacking a skilled and educated workforce, reliance on labour-intensive methods, and the lack of unified regulatory mechanisms to promote safety culture [24,25]. Such inhibiting elements negate the development of a positive safety culture, increasing risks and accidents and affecting SMEs’ construction performance [26].

### 1.1. Safety Culture among Construction SMEs in Ghana

A series of studies have shed light on the safety culture among construction SMEs in Ghana. For example, a health and safety (H&S) compliance model was developed for construction SMEs in Ghana [27]. Barriers to developing construction SMEs in Ghana include financial, business development, technical, corruption, and knowledge management barriers [28]. The capacity of SMEs is further affected by factors like delay in payment, limited access to finance, non-payment of interest on delayed payments, and a lack of fair competition [29]. The challenges are exacerbated by delays in payment, politics/corruption in contract awards, and difficulties in accessing credit [30]. Despite these challenges, there needs to be a higher level of application of lean construction principles among SMEs in Ghana [31]. Entrepreneurial features, firm characteristics, the business environment, and stakeholder involvement can drive the growth of these firms [32,33]. The health and safety (H&S) practices of SME contractors in the Ghanaian construction industry are a significant problem [27]. Several studies have highlighted challenges and improvement areas [9,34]. A framework for adaptive capacity implementation has been proposed to address these issues, focusing on education and training, government support, and a centralised information hub [33].

These SMEs’ challenges include low literacy levels, economic constraints, and a need for safety consciousness [9,14]. SMEs’ organisational culture and safety practices, emphasising organisational culture for safety compliance, have been examined [27]. Others have explored the factors influencing safety management and the implementation of health and safety policies, with the latter suggesting the need for a regulatory body [16,19]. Another study provided a cultural perspective, with the former identifying the influence of local cultures on risk-taking behaviour and the latter proposing knowledge transfer from construction companies to communities [35]. All these studies collectively underscore the need for a more proactive approach to safety management and integrating local cultures in safety education and training. From the above, it is evident that a framework for a positive safety culture has yet to be developed in Ghana to help construction SMEs establish a positive safety culture outlook to enhance safety performance.

### 1.2. Positive Safety Culture

A positive safety culture in the construction industry is crucial for improving H&S performance. It is characterised by continuous improvement and the ability to anticipate H&S risks [36]. Safety leadership is vital in enhancing safety culture, focusing on leadership, safety behaviour, planning, individual capabilities, and reporting [37]. The construction safety culture significantly influences safety behaviour and motivation [38]. A safety culture index, including management commitment, accountability, worker involvement, supervisory leadership, communication, and safety education and training, can be used to measure safety culture [39]. However, there are challenges in implementing a safety culture, such as more awareness about contractual provisions and safety training and education [40]. Management commitment, safety procedures, and compliance significantly affect safety culture [41]. A positive safety culture in the construction industry is characterised by strong management commitment, effective safety procedures, and high safety compliance [41]. This resilient culture focuses on safety leadership and competency [36,42]. It is shaped by a robust H&S supervision system, emphasising leadership, policy, safety planning, safety and health committees, and training [43]. In high-rise construction, safety culture is influenced by factors such as safety supervision and training [44]. However, more safety practices must be implemented, such as contractual provisions and safety training [45]. The conduct of the construction workforce is also a key factor, which can be shaped through a psychological contract of safety [46].

### 1.3. Characteristics of Positive Safety Culture

A high frequency of health and safety (H&S) missteps within construction SMEs suggests that leadership needs more commitment [47]. This indicates a need to foster a positive safety culture to decrease workplace incidents [2,48]. A strong safety culture leads to better employee performance and adherence to safety regulations [49]. The absence of explicit health and safety legislation in Ghana heightens the importance of embedding a strong safety culture in construction SMEs. A positive safety culture, characterised by prioritisation, employee engagement, open communication, and integration into organisational ethos, is essential for improving Health and Safety outcomes [2]. Understanding psychological, behavioural, and contextual factors is necessary to assess safety culture’s impact on safety outcomes [36].

Measuring safety culture, while challenging, is crucial for tangible improvements and can be achieved through specific methodologies and tools [50]. Essential elements such as management concern, trust, and continuous improvement are central to a positive safety culture, which can be cultivated through commitment, employee involvement, and strategic campaigns [51,52]. Management involvement and adaptability are integral to assessing safety culture [53]. The authors of [53] conceptualised adaptability, information dissemination, behavioural standards, commitment to safety, and safety awareness as primary measures to promote safety culture within construction SMEs.

However, the willingness of management and staff to learn from historical experiences and make informed decisions about safety culture towards enhancement of construction safety procedures for SME construction firms still needs to be included. Developing a safety model that promotes a safety culture through safety-related information sharing, safety adaptation, safety behaviour, safety awareness, and a safety commitment among SMEs in the construction space is critical. The assessment examined how deeply ingrained safety values are shaped through safety behaviour within the SME construction firms, measuring the SMEs-wide dedication to safety and the positivity on safety issues to improve overall SME construction performance.

### 1.4. Safety Culture Models

The construction industry has seen the development of various safety culture models to address its inherent risks and improve safety management practices. One essential advancement is the maturity model for resilient safety culture in construction companies [36]. This model characterises a resilient safety culture capable of managing changing and unforeseen health and safety risks, which are common due to the complexity of construction projects. The model outlines five maturity levels and provides a set of descriptors for 19 aspects of a resilient safety culture at each level, offering a pathway for organisations to benchmark and improve their safety risk management capabilities. In addition, SMEs focus on creating a clear plan towards achieving safety excellence through the advancement of a positive safety culture has been advanced. The elements that predict safety culture include adaptability, information dissemination, behavioural standards, commitment to safety, and safety awareness to promote safety culture within construction SMEs.

## 2. Hypotheses Development

### 2.1. Safety Commitment to the Safety Culture of Construction SMEs

Commitment to safety is described as individual involvement in health and safety activities within an organisation to achieve safety goals that will improve safety performance in construction SMEs [54]. Commitment to safety in construction SMEs is a shared responsibility between the workforce and management. It can be argued that responsibility can only be established if a positive safety culture exists among construction SMEs. According to [54], the behaviour and attitudes of employees contribute to safety commitment, which directly impacts safety culture. The degree to which construction SMEs at all levels have a favourable attitude towards safety is reflected in their commitment to safety. The authors of [54] indicated that the behaviour and attitude of employees usually demonstrate the level of commitment to safety. When employees of construction SMEs are committed to safety, they behave safely on site, not only because they perceive hazards, but they instead do so because safety has become a culture among them [55]. When there is a commitment to safety, there is teamwork and collaboration between construction employees to prevent accidents, injuries, and fatalities. The study hypothesises that a high commitment to safety culture significantly influences SMEs’ construction practices during work.

### 2.2. Safety Behaviour in the Safety Culture of Construction SMEs

Safety behaviour is pivotal in shaping the safety culture within small and medium-sized enterprises (SMEs) operating in the construction industry. A robust safety culture is essential for ensuring the workforce’s well-being and mitigating the risks associated with construction activities. According to the Domino Theory, the two most important elements contributing to accidents are unsafe employee behaviour and unsafe object circumstances [56]. According to [56], eight significant causes of construction site accidents are a lack of safety equipment, a lack of appropriate training, deficient safety enforcement, unsafe site conditions, unsafe methods of carrying out work, isolated deviation from prescribed behaviour, and a poor safety attitude of not using safety equipment. Effective safety programs are essential for identifying and remediating hazards in the workplace. However, construction SMEs need health and safety intervention to establish a positive safety culture, thereby eliminating behavioural tendencies to safety protocols often exhibited at sites. This means a positive safety culture and the safe behaviour of workers on sites for SMEs may be related. Ref. [57] assessed safety management factors to develop a research agenda for the construction industry. Critical components of safety programs such as hazard identification, hazard analysis, and hazard remediation strategies (Gan 2019) are relevant in fostering a positive safety culture among construction SMEs. The supervisor’s safety behaviour, management commitment, safety behaviour, co-worker safety behaviour, and the psychological agreement to be safe [57,58] are critical in safety culture and values. A positive safety culture within construction SMEs may predict safety-related behaviours. The study hypothesises that safety behaviour in the safety culture will significantly influence SMEs’ construction.

### 2.3. Safety Awareness on the Safety Culture of Construction SMEs

Safety awareness is the ability of employees to judge and identify dangers in purposeful production activities [59]. Awareness of an unsafe factor is what the human brain has on objective existence [60]. The unsafe behaviour of employees, the unsafe state of things, and the environmental response to hazardous conditions may be attributed to a poor safety culture. The commitment to the health and safety of construction SMEs depends on the level of awareness of employees of health and safety [61,62]. A low level of safety awareness implies a negative safety culture. Within a positive safety culture, workers usually develop an awareness of site safety, recognising that safety is paramount in every activity they undertake [63]. Safety awareness measures how well the management and staff of construction SMEs are aware of the risks to their safety and the safety of others related to business operations. Safety awareness allows employees to recognise a wide range of hazards and the effects of individual actions in the workplace, eliminating job-induced risks [64]. The study hypothesises that safety awareness in the safety culture will significantly influence SMEs’ construction.

### 2.4. Safety Information on the Safety Culture of Construction SMEs

Safety information refers to the extent to which safety information is disseminated among management and employees of an organisation at the right time and among the right people [65]. Sharing safety information is the bridge that connects the behaviour of both management and workers of construction SMEs [62]. Safety information includes employers sharing information that most often indicates the safety status of a particular activity with employees within an organisation [66,67]. Employers must ensure that resources on safety management and clear policy designs on safety culture are available and communicated so the workforce can act swiftly. Therefore, it is appropriate to suggest that injuries, accidents, and fatalities occur only when there is a failure to share safety information on safety culture. Everyone has responsibility, whilst employees of construction SMEs should be encouraged to communicate safety-related information at the right time among themselves at sites; management (employers) must also show leadership by ensuring appropriate design policies (training) and resources are in place for people on construction sites to avoid hazardous situations that lead to accidents. Workers are protected when they adhere to management instructions and training on health safety culture. Complete, timely and accurate safety information is the bedrock of a positive safety culture [68]. Hence, owners of construction SMEs must pay special attention to the accuracy and timeliness of safety information across the length and breadth of their companies. Establishing mechanisms to communicate safety information within construction SMEs to support a robust and positive safety culture becomes relevant [69]. The study hypothesizes that Safety Information on the Safety Culture will significantly influence Construction SMEs

### 2.5. Safety Adaptability on the Safety Culture of Construction SMEs

Adaptability is an individual’s cognitive, behavioural, and emotional regulation or adjustment during change and uncertainty [70]. Adaptability empowers employees and management of construction SMEs to anticipate problems, keep up with changes, embrace new ways of doing things, cope with emergencies, and adjust swiftly to alterations. Adaptability enables workers in construction SMEs to respond effectively to changes and different demands on construction sites [71]. The adaptability of employees of construction SMEs to safety practices is one of the surest means to reduce the occurrences of accidents among construction SMEs. The factors that prevent workers in construction SMEs from implementing positive health and safety practices and adaptation of employees to safety procedures are against their traditional training, working conditions that are unethical due to human psychological quirks, inadequate guidance regarding the working conditions, site safety managers providing insufficient and inefficient supervision, and unsafe practices by workers because of religious assertions [72]. The lack of adaptability to safety practices on construction sites is the leading cause of construction SMEs’ injuries, accidents and fatalities [71,73]. Safety adaptability plays a significant role in preventing accidents on construction sites of construction SMEs and promoting a positive safety culture. The study hypothesizes that Safety Adaptability on the Safety Culture will significantly influence Construction SMEs

**Figure 1:**
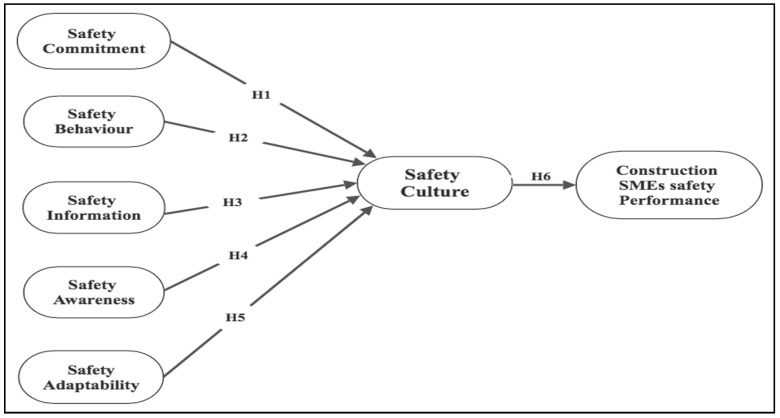
Conceptual framework for a positive safety culture for construction SMEs. Source: author’s construct.

The relationship between safety behaviour, safety commitment, safety awareness, safety information, and adaptability to construction SMEs’ safety culture is multifaceted and crucial for developing a robust safety culture (Figure 1). This improves overall performance and significantly impacts the overall safety environment within construction SMEs. Behaviour related to safety compliance, risk identification, reporting, and communication establishes a safety culture in construction workplaces. These behaviours are driven by individuals and organizations, which ensure personal commitment and organizational policies that foster a safe culture. Safety management practices significantly influence safety outcomes in SMEs adaptable to safety measures. The safety knowledge resulting from safety awareness influences safety behaviour, enhancing safety information and creating a more robust safety culture.

## 3. Material and Methods

### 3.1. The Participants

The study involved 31 health and safety experts from Ghana, who all initially agreed to participate in a Delphi survey process. However, by the end of the third round, only 16 had completed the study, supporting existing literature that participant dropout rates increase with the number of Delphi rounds due to factors like fatigue and cost [74]. The experts were from various major cities in Ghana, making the study notable for its geographical diversity and the balance between academic and practical perspectives. Most experts were male, with most holding master’s degrees or higher. The representation adopted from [2] included academics and practitioners in the health and safety field.

### 3.2. Materials

A Delphi survey involving three rounds was conducted with a panel of 31 health and safety experts in Ghana, out of whom 16 completed the entire process. Following the final round, a consensus was reached on 79 of the 87 leading indicator metrics identified during a literature review, which were retained. The agreed indicators were then transformed into a questionnaire format, utilizing a 5-point Likert Scale, and distributed to construction SMEs across Ghana. Table 1 identifies the indicators measured by safety behaviour, safety commitment, safety awareness, safety information, and safety adaptability to the safety culture of construction SMEs.

### 3.3. Procedure

The sampling process for this study was initiated by contacting the Building and Civil Engineering Contractors Association of Ghana to secure member contractors’ emails and phone numbers nationwide. Members were contacted via phone to gauge their willingness to participate in the study, followed by distributing the questionnaires through emails and a drop-and-collect method. Additionally, in over 200 construction sites across different regions, questionnaires were handed out to the top management of each construction firm using a drop-and-collect system. To ensure a high response rate, follow-up calls and reminder emails were sent, urging respondents to complete and return the questionnaires by the specified deadline between June 2019 and October 2022. In total, 450 questionnaires were distributed—350 through drop-and-collect and 100 via email. Of these, 284 were returned, yielding a response rate of 63.11%, representing the study’s sample size. The collected responses from management were analysed to assess the impact of each component on fostering a positive safety culture within the industry.

### 3.4. Data Analysis

#### 3.4.1. Measurement Model Assessment

We first assessed the measurement model, which focuses on the validity and reliability of latent variables [87]. Thus, the results of the reliability and convergent validity of the measures were examined and reported in Table 2. This was followed by discriminant validity assessment using the Heterotrait-Monotrait (HTMT) ratio [87] reported in Table 3. The results in Table 2 prove that all the constructs had acceptable construct reliability and convergent validity levels. Explicitly, each construct had reliability values above 0.70 (Cronbach alpha ranges from 0.728 to 0.903; rho ranges from 0.757 to 0.912; rho_c varies from 0.844 to 0.923), and the AVEs were more than the threshold value of 0.50 (ranging from 0.533 to 0.706). Thus, it was evident that all the constructs displayed fitting construct reliability and convergent validity [87]. Discriminant validity of the constructs reported using HTMT values was also established as the values were below the cut-off value 0.90. This established that all variables were distinct, a necessary condition for discriminant validity [87].

#### 3.4.2. Structural Framework Assessment (Hypotheses Testing)

**Figure 2:**
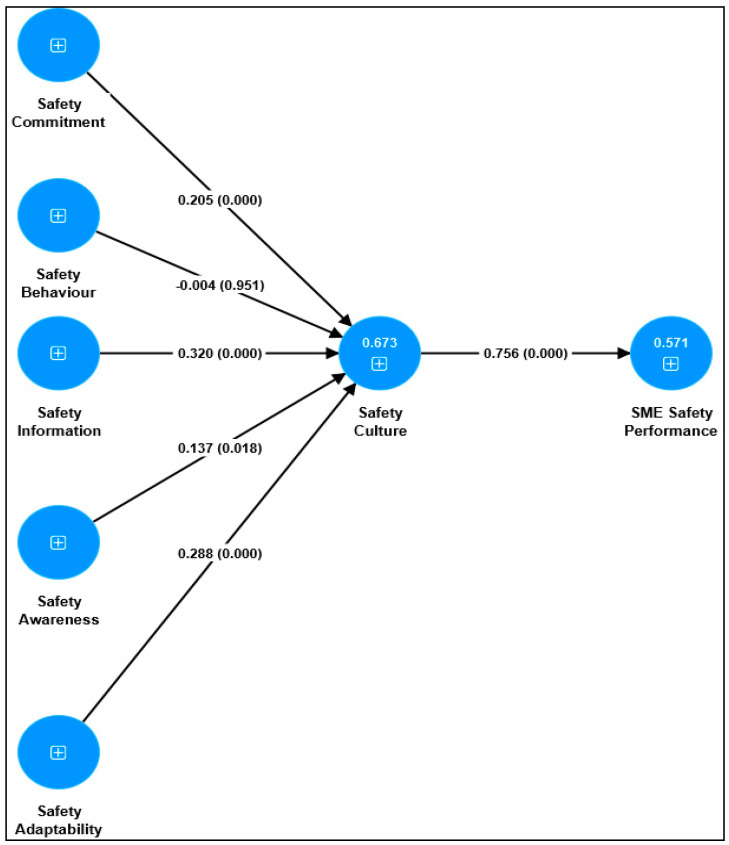
Structural framework results.

Having established adequate reliability and validity of the measurement framework to establish the consistency, accuracy and credibility of data and findings [87]. Partial least-squares structural equation modelling (PLS-SEM) analysis assessed the structural paths, including the multi-collinearity checks, direct and indirect effects, model fit, and explanatory power [88]. Structural Framework Assessment indicates the relationships of bootstrapping to derive standard errors and confidence intervals for significance testing and interpret results in the context of safety culture, focusing on the predictive relevance and practical implications. The results, which are presented in Table 2 and Figure 2, were obtained from a bootstrapping procedure (5000 sub-samples) [87,89].

As presented in Table 4, the results demonstrated no multi-collinearity concerns, as all the Variance Inflation Factor (VIF) values were below 3, ranging from 1.000 to 2.765 [89]. Furthermore, the structural framework exhibited a good fit, as the Standardized Root Mean Squared residual (SRMR) values were less than 0.08 [90]. The R^2^ values revealed that the structural model explained 57.1% and 67.3% of the variation in safety performance and safety culture, respectively. Thus, the model demonstrated substantial explanatory power in predicting safety performance and safety culture [89].

Five of the six direct paths (i.e., hypotheses) were significantly positive regarding the direct effects. Precisely, as demonstrated in Table 2 and Figure 2, safety commitment (*β* = 0.205; *SE* = 0.053; *t* = 3.846; *p* = 0.000; 95% CI [0.106; 0.312]) and safety information (*β* = 0.320; *SE* = 0.056; *t* = 5.700; *p* = 0.000; 95% CI [0.210; 0.430]) positively influenced safety culture significantly. Similarly, safety awareness (*β* = 0.137; *SE* = 0.058; *t* = 2.373; *p* = 0.018; 95% CI [0.021; 0.246]) and safety adaptability (*β* = 0.288; *SE* = 0.054; *t* = 5.348; *p* = 0.000; 95% CI [0.181; 0.392]) positively influenced safety culture significantly. However, safety behaviour (*β* = −0.004; *SE* = 0.060; *t* = 0.061; *p* = 0.951; 95% CI [−0.116; 0.113]) failed to predict safety culture significantly positively. Thus, hypotheses H1, H3, H4, and H5 were accepted. The last hypothesis (i.e., H6) was also supported because safety culture (*β* = 0.756; *SE* = 0.028; *t* = 27.180; *p* = 0.000; 95% CI [0.692; 0.804]) positively influenced safety performance significantly.

Except for safety behaviour, the results revealed further significant indirect effects of safety commitment (*β* = 0.155; *SE* = 0.041; *t* = 3.767; *p* = 0.000; 95% CI [0.072; 0.231]), safety information (*β* = 0.242; *SE* = 0.042; *t* = 5.738; *p* = 0.000; 95% CI [0.162; 0.326]), safety awareness (*β* = 0.104; *SE* = 0.044; *t* = 2.361; *p* = 0.018; 95% CI [0.016; 0.187]), and safety adaptability (*β* = 0.218; *SE* = 0.042; *t* = 5.162; *p* = 0.000; 95% CI [0.140; 0.304]) on safety performance through safety culture. These results suggest that safety culture mediates the influence of safety commitment, information, safety awareness, and adaptability on safety performance. Thus, considering both significant direct and indirect effects, safety culture partially mediates the impact of safety commitment, safety information, safety awareness, and adaptability on safety performance [91,92].

## 4. Results

Five themes were identified regarding the performance of construction SMEs: safety commitment, safety information, safety adaptability, safety awareness, and safety behaviour. See Table 3, published in [2], for the combined Delphi survey results, which are R1, R2, and R3 of importance.

## 5. Discussion

The first theme identified was the commitment to safety and the culture of construction SMEs. The commitment to safety within construction SMEs in Ghana significantly enhances a positive safety culture, reducing injuries, accidents, and fatalities. This finding aligns with the authors, who have argued that safety commitment behaviours are crucial in lowering accident rates on construction sites [75]. Additionally, the data emphasised that practical safety commitment in construction SMEs should be visible through support and investment in site safety, which fosters a positive safety culture [55]. Hence, implementing leading safety indicators effectively is critical to improving safety performance among construction SMEs in Ghana.

The result found that the availability and effective dissemination of safety information significantly enhances the safety culture within construction SMEs. This positive impact on safety performance is because complete, timely, and accurate safety information is crucial for establishing a solid safety culture in these companies [93]. Furthermore, developing mechanisms to communicate safety information effectively within the construction industry, particularly among construction SMEs, fosters a robust and positive safety culture [69]. The study highlights the significant correlation between safety adaptability and a positive safety culture within construction SMEs. As detailed by a previous study, construction workers exhibit adaptability and flexibility, which are crucial in facilitating their tasks effectively [94]. This adaptability not only enhances the dedication of these workers towards health and safety (H&S) but also bolsters their well-being. Consequently, adaptability is pivotal for fostering an influential safety culture, implying that the quicker the construction workforce assimilates safety practices, the safer their working environments become. This research underpins strategies for SME owners and managers to cultivate a safety-oriented culture that ultimately elevates health and safety standards in the construction sector.

The study indicates a significant positive relationship between safety awareness and a positive safety culture within construction SMEs. This is supported by the literature, which confirms that increased safety awareness among employees positively impacts the safety culture in these organisations [59,61]. It also notes that a lack of safety awareness and insufficient knowledge of safety measures significantly contributes to injuries, accidents, and fatalities in the construction sector. Thus, enhancing safety awareness is crucial for fostering a positive safety culture in construction SMEs, ultimately leading to reduced risks and better safety outcomes in the industry. However, the relationship between safety behaviours and safety culture was found to be negative rather than positive, leading to non-acceptance of the hypothesis. It is observed that employees’ behaviours and attitudes significantly influence their commitment to safety, impacting the safety culture [54]. Several scholars have reported that human-related factors, such as unsafe behaviours of construction workers, account for approximately 70% of onsite accidents [95]. They further emphasised that such dangerous behaviours are the primary causes of accidents, injuries, and fatalities in the construction industry. Furthermore, risky behaviours by construction employees are significant contributors to accidents and fatalities in construction SMEs [96,97]. Some schools of thought say dangerous behaviours on construction sites alone are responsible for about 80% of accidents [98]. However, in recent years, industry and academia have realised that accident causations are systematic and not limited to human behaviour. The outcome of this hypothesis can thus be viewed in this light.

The positive and significant causal link between safety culture (intervening variable) and safety performance (dependent variable) affirmed the hypothesis. This correlation is supported by scholars’ research, who have confirmed that safety culture substantially influences safety performance [99,100]. For instance, a study noted that a robust positive safety culture in construction SMEs could prevent 98% of injuries, accidents, and fatalities, noting that a lack of such culture leads to errors and violations of safety rules, highlighting management’s failures in safeguarding employees [101]. Furthermore, safety performance could be improved through proactive measures, such as assessing the safety climate and culture and recognising potential hazards, or through reactive measures, focusing on injury, accident, and fatality rates and the costs of compensating accident victims [102]. These measures are crucial indicators for enhancing safety performance. Thus, construction SMEs in the Ghanaian construction industry need to develop a robust and positive safety culture by integrating the core elements and leading indicators outlined in this research to improve their safety performance.

Finally, a Spearman correlation matrix was utilised to examine the relationships between demographic variables and various safety-related factors, such as safety commitment, safety information, safety adaptability, safety awareness, safety behaviour, and overall safety culture and performance in construction SMEs. The analysis highlighted a significant correlation between the role of the officer (management) in charge of health and safety (H&S) and the safety commitment of employees. Further findings indicate that this officer’s role critically affects how safety information is disseminated, how adaptable workers are to safety protocols, their behaviour towards safety and colleagues, their awareness of safety issues, a positive safety culture, and the overall safety performance within these SMEs. This suggests that appointing dedicated health and safety (H&S) officers in management positions could enhance the safety culture and improve performance in construction SMEs. The implication is that these safety officers in SME management positions can design and formulate policies on safety culture and provide needed resources in mitigating risk at construction sites. These findings have been cited in the literature, posing that commitment, behaviour, information, adaptability, and awareness are essential to fostering a positive safety culture in construction [65,103,104]. It is recommended that construction SMEs focus on demographic variables that positively influence these critical elements to bolster safety performance.

## 6. Limitations

The research was conducted only in major cities of Ghana due to the concentration of construction SMEs in these areas, making it logistically unfeasible to extend the study across all towns and rural areas. Further, the methodological constraint was the inability to add more items or constructs that could have enhanced the reliability and validity of the study. In particular, a questionnaire survey inherently restricts deeper inquiry into the participants’ responses. Incorporating interviews that elicit worker inputs could have allowed for a more detailed exploration of reactions, enhancing the study’s depth.

Further, the occurrence of non-significant correlations among some of the constructs limited the study, leading to the non-acceptance of a hypothesis. The study focused on a limited number of health and safety (H&S) management practices and top management commitment to safety culture for positive safety culture intuitions in construction SMEs. Moreover, potentially useful leading indicator metrics should have been included following the Delphi survey due to a lack of consensus. The framework adopted is a work in progress which could be used in later research. The inclusion of more comprehensive techniques, such as direct observations or interviews with worker inputs from construction SMEs, could provide more nuanced insights into critical safety practices.

## 7. Conclusions

A literature review identified five critical elements, safety commitment, safety behaviour, safety awareness, safety information, and safety adaptability, foundational to fostering a positive safety culture in construction SMEs. The review suggests that inadequate management commitment, behaviour, awareness, information dissemination, and adaptability towards safety culture are prevalent in these organisations, leading to a higher incidence of accidents. It was further highlighted that establishing a positive safety culture is essential for reducing accident occurrences within construction SMEs in Ghana. The primary data from the research revealed that four of the five critical components of safety commitment, safety awareness, safety information, and safety adaptability positively impact the development of a positive safety culture within construction SMEs in Ghana. It was emphasised that construction SMEs could establish and maintain a positive safety culture when these factors are considered in their development strategies to enhance safety performance. These components have been integrated into a framework to cultivate a positive safety culture in the sector.

The surprising outcome of this study, which found that safety behaviour does not substantially affect safety culture among construction SMEs in Ghana, can be rationalised through several key considerations:

- Firstly, it is crucial to acknowledge the unique characteristics of the construction industry in Ghana, which significantly differ from those in other regions where similar studies have been conducted. This industry in Ghana is often marked by a high degree of informality, potentially leading to inconsistent enforcement and adherence to established safety protocols, thereby diminishing the impact of individual safety behaviours on the overall safety culture.
- Additionally, the influence of societal and cultural norms prevalent in Ghana cannot be overlooked. Ghanaian society’s collectivist nature suggests that community and group dynamics may significantly shape safety culture more than individual safety behaviours.
- Furthermore, in Ghana, where respect for authority is deeply ingrained, leadership and management practices likely have a more substantial impact on safety culture than the behaviours of individual workers.
- The construction sector’s project-based nature, characterised by a temporary and fluid workforce, suggests that safety culture may be unstable and less influenced by consistent safety behaviours over time.
- Finally, it is essential to consider the limitations of the measurement tools used in this study. While these tools have been validated in other contexts, they may not fully capture specific local cultural nuances or industry-specific details.

Given these factors, this study’s absence of a significant link between safety behaviour and safety culture highlights the need for further investigation to fully understand the specific dynamics of safety culture within Ghana’s construction industry. This finding also underscores the importance of contextual and cultural factors when extrapolating results to different settings. The study demonstrated that construction SMEs could enhance their safety performance by establishing a positive safety culture within their organisations. This relationship between a positive safety culture and improved safety performance is positive and statistically significant. Further insights from the Delphi survey identified 79 leading indicator metrics, categorised into 14 core elements, as essential for construction SMEs to adopt to foster a positive safety culture and boost safety performance. The survey conducted among construction SMEs corroborated the importance of these leading indicators in developing a positive safety culture that significantly improves safety outcomes in Ghanaian construction SMEs. It is firmly accepted that construction SMEs that establish a positive safety culture can potentially reduce accidents and injuries.

In conclusion, a robust safety culture is significantly influenced by the interplay of safety behaviour, safety commitment, safety awareness, safety information, and adaptability to the safety culture of construction SMEs. Effective safety behaviour practices are rooted in the responsibility of both management and workers to prioritise safety, which fosters a heightened awareness of safety protocols and potential hazards. The dissemination and accessibility of clear and comprehensive safety information enhance this awareness, providing the necessary knowledge for safe practices. Finally, the ability to adjust and adapt to safety measures and behaviour in response to evolving work conditions and new safety challenges ensures the continuous improvement and resilience of the safety culture among Ghanaian SMEs in the construction industry.

## Figures and Tables

**Table 1:** Positive safety culture constructs and their respective core elements.

Constructs	Core Elements	References
Commitment	Management concerns	[75,76]
	Safe work procedures	[77]
Information	Reporting	[78]
	Information Sharing	[62]
Adaptability	Adaptability and flexibility	[79,80]
	Collective responsibility	[81]
Awareness	Equipment and plant	[82]
	Hazard and risks	[83]
	Education and training	[84,85]
Behaviour	Worksite norms	[21]
	Job satisfaction	[86]

**Table 2:** Reliability and convergent validity results.

Constructs	Cronbach’s Alpha	Composite Reliability (rho_a)	Composite Reliability (rho_c)	Average Variance Extracted (AVE)
Safety Adaptability	0.883	0.887	0.909	0.588
Safety Awareness	0.728	0.757	0.844	0.644
Safety Behaviour	0.769	0.798	0.850	0.588
Safety Commitment	0.903	0.912	0.923	0.600
Safety Information	0.822	0.842	0.871	0.533
Overall Safety Culture	0.861	0.861	0.906	0.706
SME Safety Performance	0.847	0.859	0.887	0.568

**Table 3:** Discriminant validity by HTMT criterion.

Constructs	1	2	3	4	5	6	7
SME Safety Performance							
2. Safety Adaptability	0.812						
3. Safety Awareness	0.827	0.721					
4. Safety Behaviour	0.803	0.809	0.847				
5. Safety Commitment	0.792	0.697	0.792	0.759			
6. Safety Culture	0.873	0.816	0.801	0.757	0.778		
7. Safety Information	0.859	0.794	0.838	0.856	0.777	0.869	

**Table 4:** Structural model results.

Paths	Β	SE	*t*-Statistics	*p*-Values	VIF	Confidence Interval	
						2.5%	97.5%
Direct Effects							
SCom => SC	0.205	0.053	3.846	0.000	2.327	0.106	0.312
SB => SC	−0.004	0.060	0.061	0.951	2.490	−0.116	0.113
SI => SC	0.320	0.056	5.700	0.000	2.765	0.210	0.430
SAw => SC	0.137	0.058	2.373	0.018	2.178	0.021	0.246
SA => SC	0.288	0.054	5.348	0.000	2.358	0.181	0.392
SC => SP	0.756	0.028	27.180	0.000	1.000	0.692	0.804
Indirect Effects							
SCom => SC => SP	0.155	0.041	3.767	0.000	-	0.072	0.231
SB => SC => SP	−0.003	0.045	0.061	0.952	-	−0.085	0.090
SI => SC => SP	0.242	0.042	5.738	0.000	-	0.162	0.326
SAw => SC => SP	0.104	0.044	2.361	0.018	-	0.016	0.187
SA => SC => SP	0.218	0.042	5.162	0.000	-	0.140	0.304
Model’s Summary							
**Constructs**	**R^2^**	**R^2^ adjusted**		**Framework Fit using SRMR**			
SP	0.571	0.570		Saturated framework			0.059
SC	0.673	0.667		Estimated framework			0.075

SB = safety behaviour; SAw = safety awareness; SCom = safety commitment; SI = safety information; SA = adaptability; SC = safety culture; SP = safety performance; SE = standard error; VIF = Variance Inflation Factor.

## Data Availability

The primary data supporting the conclusions of this article will be made available by the corresponding author upon request.
